# Cerebral Blood Flow, Heart Rate, and Blood Pressure Patterns during the Tilt Test in Common Orthostatic Syndromes

**DOI:** 10.1155/2016/6127340

**Published:** 2016-07-20

**Authors:** Peter Novak

**Affiliations:** Department of Neurology, Brigham and Women's Faulkner Hospital, Harvard Medical School, Boston, MA 02130, USA

## Abstract

*Objective*. The head-up tilt test is widely used for evaluation of orthostatic intolerance. Although orthostatic symptoms usually reflect cerebral hypoperfusion, the cerebral blood flow velocity (CBFv) profile in orthostatic syndromes is not well described. This study evaluated CBFv and cardiovascular patterns associated with the tilt test in common orthostatic syndromes.* Methods*. This retrospective study analyzed the tilt test of patients with history of orthostatic intolerance. The following signals were recorded: ECG, blood pressure, CBFv using transcranial Doppler, respiratory signals, and end tidal CO_2_.* Results*. Data from 744 patients were analyzed. Characteristic pattern associated with a particular orthostatic syndrome can be grouped into abnormalities predominantly affecting blood pressure (orthostatic hypotension, orthostatic hypertension syndrome, vasomotor oscillations, and neurally mediated syncope—cardioinhibitory, vasodepressor, and mixed), cerebral blood flow (orthostatic hypoperfusion syndrome, primary cerebral autoregulatory failure), and heart rate (tachycardia syndromes: postural tachycardia syndrome, paroxysmal sinus tachycardia, and inappropriate sinus tachycardia). Psychogenic pseudosyncope is associated with stable CBFv.* Conclusions*. The tilt test is useful add-on in diagnosis of several orthostatic syndromes. However diagnostic criteria for several syndromes had to be modified to allow unambiguous pattern classification. CBFv monitoring in addition to blood pressure and heart rate may increase diagnostic yield of the tilt test.

## 1. Introduction

The head-up tilt testing is widely used in diagnosis of orthostatic intolerance. The tilting provokes blood pooling in the splanchnic and the lower extremity circulation which triggers the neural and humoral compensatory mechanisms aiming to maintain stable blood pressure and cerebral perfusion. The tilt test assesses the integrity of parasympathetic and sympathetic innervation [1]. The tilt test was initially introduced to diagnose vasovagal syncope [2]. Since then the diagnostic utility of the tilt test has been expanded for diagnosis of orthostatic hypotension, autonomic failure, postural tachycardia syndrome, and other forms of orthostatic intolerance [3].

Common orthostatic symptoms are usually defined by heart rate and blood pressure responses to tilting. The orthostatic cerebral blood flow is less studied; even many orthostatic symptoms are due to orthostatic cerebral hypoperfusion [4]. Therefore, the purpose of this study was to analyze the cerebral blood flow and cardiovascular patterns in common orthostatic syndromes.

## 2. Methods

*Standard Protocol Approvals, Registrations, and Patient Consents*. The study was approved by the Institutional Review Board of the University of Massachusetts Medical School as a minimal risk study.

### 2.1. Participants

This retrospective, single-center study included patients with history of autonomic function testing at the University of Massachusetts Medical School, Autonomic Laboratory, between years 2007 and 2014. Patients were referred for evaluation of two types of orthostatic complaints. The first group constitutes orthostatic intolerance symptoms which include dizziness, lightheadedness, unexplained falls, chronic fatigue, heaviness, chest pain, and shortness of breath. The second group includes patients with history of unexplained loss of consciousness that were referred for evaluation of suspected syncope.

### 2.2. Autonomic Cardiovascular Testing

The details of our standardized testing protocol have been published previously [5]. Autonomic testing included deep breathing, Valsalva maneuver, and the tilt test. Only the tilt test results are reported here. After a resting supine period of at least 10 minutes duration, subjects were tilted at 70 degrees for 10 minutes or longer if patients can tolerate. During the whole testing, heart rate, blood pressure, respiratory movement, and cerebral blood flow velocity (CBFv) were monitored.

Heart rate was calculated from ECG. Arterial blood pressure was measured in the upper arm intermittently using an automated noninvasive oscillometric device Dinamap ProCare Monitor 100 (GE, Fairfield, CT), as well as continuously by infrared finger plethysmography (Finapress Medical Systems, Amsterdam, Netherlands). Nasal thermistor and end tidal CO_2_ (Capstar-100, CWE, Inc., Ardmore, PA) were used for monitoring respiratory parameters. CBFv was monitored using transcranial Doppler (MultiDop T, DWL, Singen, Germany) with a 2 MHz probe. CBFv was obtained from the left middle cerebral artery with the Doppler probe attached to a three-dimensional positioner which kept the probe at a constant depth and angle of insonation.

The tilt responses were classified as described below.

#### 2.2.1. Normal Response

Normally heart rate increases at least 10 beats per minute (BPM) but less than 30 BMP during the tilt (Table 1) [3, 6]. Normal blood pressure responses are also restricted within a range. The normal response is a drop of systolic blood pressure <20 mmHg and diastolic blood pressure <10 mmHg on upright posture. The systolic blood pressure may normally rise up to 20 mmHg. Normal response of CBFv to the tilting is a mild drop. Since the baseline CBFv is gender and age dependent, the orthostatic criteria are given in relative values where the supine baseline is 100%. The threshold for normal drop of the mean CBFv is 90% (1st minute), 89% (5th minute), and 85% (10th minute) of the supine baseline (=100%) which immediately precedes the tilt [6].

#### 2.2.2. Postural Tachycardia Syndrome (POTS)

POTS is defined by the presence of orthostatic symptoms associated with an increment of heart rate by ≥30 BPM held for more than 30 seconds when changing position from supine to upright in the absence of orthostatic hypotension [7]. The timing of heart rate increment is either not mentioned [7] or defined within 5 minutes [8] or within 10 minutes or longer [9] of the upright posture. Early description of POTS also required the absolute heart rate 120 BPM or more, while the heart rate below 120 during the tilt test was termed as mild orthostatic intolerance [8]. The supine heart rate is not mentioned in any POTS definitions. To avoid overlap with the definition of the inappropriate sinus tachycardia (see below), an additional criterion for the POTS is average heart rate <100 BPM during supine baseline before the tilting. The CBFv may be abnormally decreased during tilting due to hyperventilation induced hypocapnia during the tilt [4]. Table 1 shows exact POTS criteria.

#### 2.2.3. Orthostatic Hypotension (OH)

Historically, OH is defined as a drop ≥20 mmHg in systolic blood pressure or ≥10 mmHg in diastolic blood pressure within three minutes of standing or head-up tilt. This definition does not take into account the baseline value; therefore a modified definition was that using a relative drop of 80% and 85% in systolic and mean blood pressure, respectively, where supine baseline is 100% [6].

#### 2.2.4. Orthostatic Hypertension Syndrome (OHTN)

OHTN is a common syndrome with a prevalence of 1.1% [10] and occurs in 16.3% of older hypertensive patients [11]. OHTN is defined as a postural increase of systolic blood pressure by at least 20 mmHg [12] or an increase in systolic blood pressure during the tilt to 120% and above where supine baseline is equal to 100% [6]. The latter definition was used in this study since it accounts for a supine baseline. Little is known about CBFv in OHTN.

#### 2.2.5. Neurally Mediated (Reflex) Syncope

Syncope is defined as a transient loss of consciousness due to global cerebral hypoperfusion [13]. Neurally mediated, also called reflex, neurocardiogenic, or vasovagal, syncope is a syncope triggered by a neural reflex resulting in systemic hypotension, reduced cardiac output, peripheral vasodilatation, and/or bradycardia [14]. Characteristic sign of neurocardiogenic syncope is profound hypotension during the tilt test. Based on the heart rate responses, syncope can be divided into three forms: cardioinhibitory, vasodepressor, and mixed which is the most common. In general, the vasodepressor syncope is due to a predominant loss of upright vasoconstrictor tone. Bradycardia or asystole predominates in cardioinhibitory syncope and both mechanisms (vasodilatation and cardioinhibition) are present in mixed syncope [3]. CBFv has characteristic pattern during syncope consistent with cerebral vasodilation as indicted by increased systolic and decreased diastolic CBFv and thus increased pulsatility index defined as systolic CBFv – diastolic CBFv [15]. In this study, the VASIS (Vasovagal Syncope International Study) classification [16] of syncope has been expanded using the CBFv and blood pressure criteria (Table 1).

#### 2.2.6. Pseudosyncope: Psychogenic Syncope

In psychogenic syncope, also termed psychogenic pseudosyncope, consciousness is only apparently lost and global cerebral hypoperfusion is absent [3]. In this study, the psychogenic syncope was defined as an apparent loss of consciousness without changes in CBFv indicative of syncope.

#### 2.2.7. Paroxysmal Sinus Tachycardia (PST)

PST can be frequently encountered during the tilt test and may be due to underlying anxiety disorder. Paroxysmal PST in this study was defined as intermittent (duration < 2 minutes) tachycardia (heart rate > 100 bpm [17]) associated with heart rate increment ≥30 BPM. The tachycardia can happen in both supine and upright positions.

#### 2.2.8. Inappropriate Sinus Tachycardia (IST)

IST is associated with persistent or recurrent elevated heart rate (>100 BMP) at rest including supine and excessive or inappropriate heart rate increment in upright position [7, 18]. In this study the “inappropriate” heart rate increment was defined as ≥30 BPM during the tilt [6].

#### 2.2.9. Orthostatic Cerebral Hypoperfusion Syndrome (OCHOs)

OCHOs is a recently described syndrome associated with reduced orthostatic cerebral blood flow velocity (CBFv) without OH, bradycardia, and excessive tachycardia [19]. OCHOs may be relatively common cause of orthostatic dizziness. Excessive tachycardia as defined in POTS is an exclusion criterion for OCHOs. OCHOs may result from cerebral vasoconstriction or abnormal venous pooling during upright position.

#### 2.2.10. Primary Cerebral Autoregulatory Failure (pCAF)

This is a new syndrome defined in this paper. PCAF is characterized by abnormally low supine CBFv without supine hypotension. The normal supine CBFv is age and gender dependent and pCAF is defined as CBFv less than the lower limit of the normal range where the normal limit is 72.09–0.38 *∗* age cm/s and 82.2–0.45 *∗* age cm/s for men and women, respectively, using the MultiDop T device (Table 1) [6]. Since this pattern (e.g., low CBFv and normal or high BP) may indicate cerebral vasoconstriction, cerebral vascular resistance defined by mean blood pressure/mean CBFv [4] has been calculated as well.

#### 2.2.11. Vasomotor Oscillations

Vasomotor oscillations are periodic fluctuations of blood pressure.

#### 2.2.12. Spurious OH due to Inaccurate Plethysmographic Blood Pressure Measurement

Blood pressure acquired by finger plethysmographic device is not always accurate [20]. It is a policy at our autonomic laboratory to acquire blood pressure by both methods, for example, oscillometric and plethysmographic, and if the sustained difference is more than 10%, then finger device should be repositioned. In the case the difference persists, blood pressure from plethysmographic device should be recalibrated using the oscillometric device.

The syndromes described above were detected automatically by an algorithm written in Matlab programming language (MathWorks, Natick, MA). The software used in this study is an extension of the Quantitative Scale for Grading of Cardiovascular Autonomic Reflex Tests and Small Fibers from Skin Biopsies (QASAT) [6], also written in Matlab. QASAT is an objective and validated instrument for grading of tilt responses which defines normal and abnormal responses in heart rate, blood pressure, and CBFv during the tilt test. Table 1 provides exact criteria for each syndrome.

### 2.3. Statistical Analysis

The clinical variables associated with orthostatic syndromes were compared with normal responses using Wilcoxon rank test since most of the data had nonnormal distribution. Nine clinical syndromes were compared with the normal response to the tilt, for example, OH, OCHOs, OHTN, POTS, IST, PST, syncope, psychogenic syncope, and pCAF, and therefore the initial significance 0.05 was adjusted by Bonferroni correction to 0.005 (0.05/10 comparisons). All statistical analyses were performed using JMP 12.0 (Cary, NC) statistical software.

## 3. Results

Data from 744 patients were analyzed. All patients had unrevealing standardized evaluation including medical history, neurological examination, basic metabolic panel, 12-lead ECG, imaging studies (CT or MRI of the brain), and EEG if patients were referred for evaluation of syncope.

669 patients were referred for evaluation of orthostatic symptoms including dizziness and 75 patients were referred for evaluation of unexplained loss of consciousness with a suspected diagnosis of syncope.

The tilt test was normal in 102 subjects (Table 2). However, from these subjects, only 7 subjects had normal entire autonomic testing (e.g., QASAT total score = 0). Remaining subjects had at least one abnormality in parasympathetic (evaluated by deep breathing test) or sudomotor (evaluated by QSART) functions.

From 669 patients evaluated for orthostatic intolerance (not syncope), the tilt test was normal in 77 patients. From 75 patients referred for evaluation of syncope, the tilt test showed normal response in 25 subjects, neutrally mediated syncope in 33 patients, and abnormal results but nonsyncope pattern in remaining patients (Table 2). Additional 23 patients that had syncope during the tilt test were referred for nonsyncope evaluation.

Table 2 shows details of orthostatic symptoms in each syndrome including the frequency of reproduction of previous symptoms that prompted autonomic testing and the frequency of a new diagnosis obtained from the tilt test. The symptoms were commonly reproduced in the PST (100%), POTS (99%), OCHOs (98%), IST (86%), and uncompensated OH (85%). Most common new diagnosis was obtained from the tilt test, which means the diagnosis was not mentioned in the chart or was not mentioned in the reason for the referral of the testing, which was OCHOs (*n* = 97), pCAF (*n* = 67), and POTS (*n* = 67).

Figures 1 [Fig fig2]–3 show common patterns encountered during the tilt testing. Figures 4 [Fig fig5] [Fig fig6] [Fig fig7] [Fig fig8] [Fig fig9] [Fig fig10] [Fig fig11] [Fig fig12] [Fig fig13] [Fig fig14] [Fig fig15] [Fig fig16] [Fig fig17] [Fig fig18] [Fig fig19] [Fig fig20]–21 show and describe relevant details of each pattern.  Figure 1 shows head-to-head comparisons of normal response, OH and OCHOs.  Figure 2 compares the profile of common tachycardia syndromes including POTS, IST, and PST.  Figure 3 shows three main types of syncope, for example, cardiovagal, vasodepressor, and mixed.

Syncope was associated with low blood pressure and CBFV (Table 2). Pure syncope pattern with otherwise normal responses on the tilt test was detected in 32 patients. Syncope was combined with other syndromes including POTS (12 patients), OHTN (1 patient), IST (3 patients), PST (3 patient), and pCAF (1 patient). Characteristic patterns of syncope are seen in Figure 3 [Fig fig4] [Fig fig5] [Fig fig6] [Fig fig7] with details in Figures 8 [Fig fig9] [Fig fig10]–11 [Fig fig12] [Fig fig13] [Fig fig14] [Fig fig15] [Fig fig16] and 17. All three types of syncope share common pattern in CBFv which is consistent with cerebral vasodilation (Figures 3 [Fig fig4] [Fig fig5] [Fig fig6] [Fig fig7] and 8 [Fig fig9] [Fig fig10]–11). Syncope can be superimposed on any pattern including IST (Figure 17).

**Figure 1:**
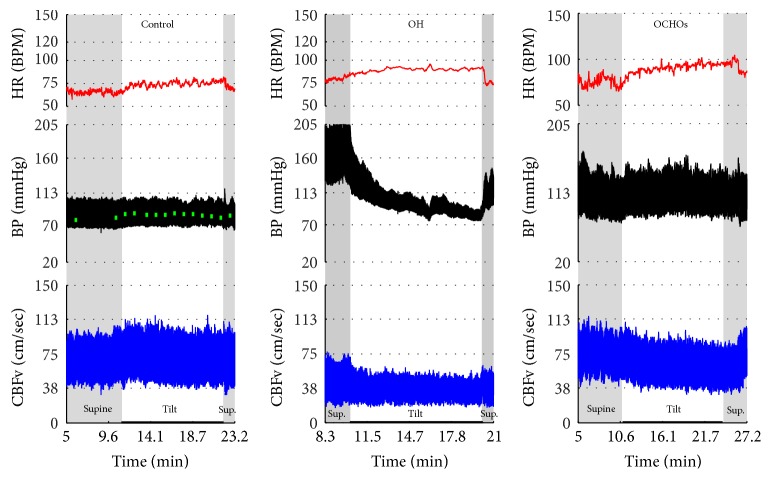
Comparisons of normal responses to the tilt (control), orthostatic hypotension (OH), and orthostatic cerebral hypoperfusion syndrome (OCHOs). Normal heart rate increment (≥10 and <30 BPM) is seen in all examples. Orthostatic blood pressure is stable in a healthy control subject and OCHOs while it is reduced in OH. Orthostatic cerebral blood flow velocity is stable in a control subject and reduced in OH and OCHOs. CBFv can be normal or reduced in OH, depending on functioning of cerebral autoregulation and severity of OH. Green boxes represent mean blood pressure obtained from the upper arm. HR = heart rate, BP = blood pressure, and CBFv = cerebral blood flow velocity.

**Figure 3 fig3:**
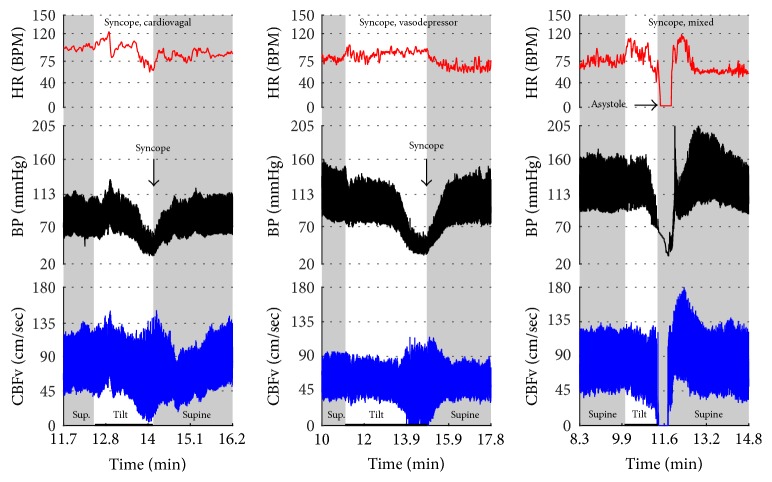
Comparisons of three main types of neurally mediated syncope. Syncope is associated with profound decline in BP and in diastolic CBFv. HR and BP responses differentiate each type of syncope while CBFv responses are similar among all syncope types. HR declines before BP in cardiovagal syncope. HR decline is absent in the vasodepressor syncope. HR and BP decline simultaneously in mixed syncope. CBFv shows typical vasodilation pattern in all types of syncope that is characterized by a decline in diastolic and increase in systolic CBFv. The diastolic CBFv is equal or close to zero during syncope.

**Figure 4 fig4:**
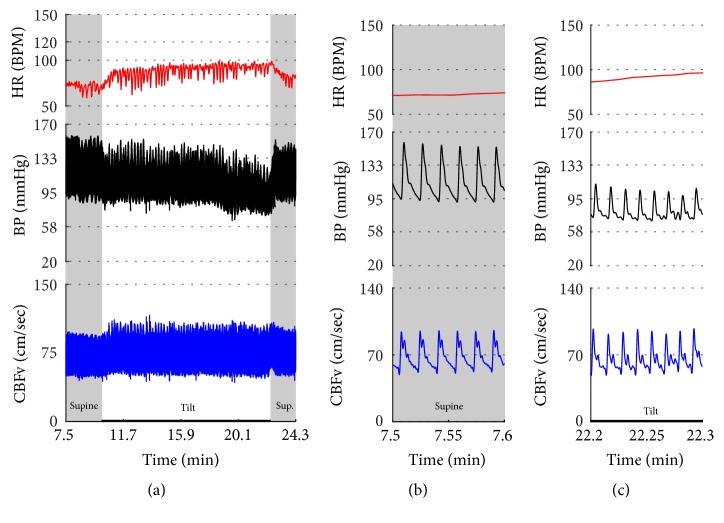
Orthostatic hypotension with stable orthostatic CBFv. There is immediate decline in BP at the onset of tilt and the decline further progressed towards the end of the tilt. The HR increment was normal and CBFv was stable during the tilt. Note details of signals in (b) and (c). Data from 60-year-old man.

**Figure 5 fig5:**
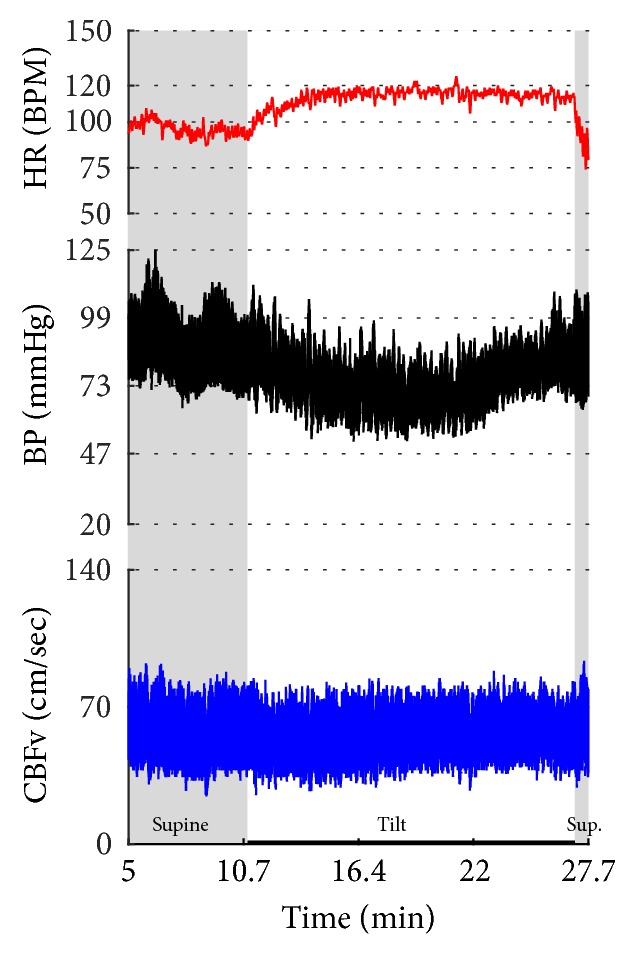
Transient orthostatic hypotension. This example shows a progressive decline in BP during the first 5 minutes of the tilt followed by the recovery of BP towards the end of the tilt. CBFv was normal and stable during the tilt. Data from 42-year-old woman.

**Figure 6 fig6:**
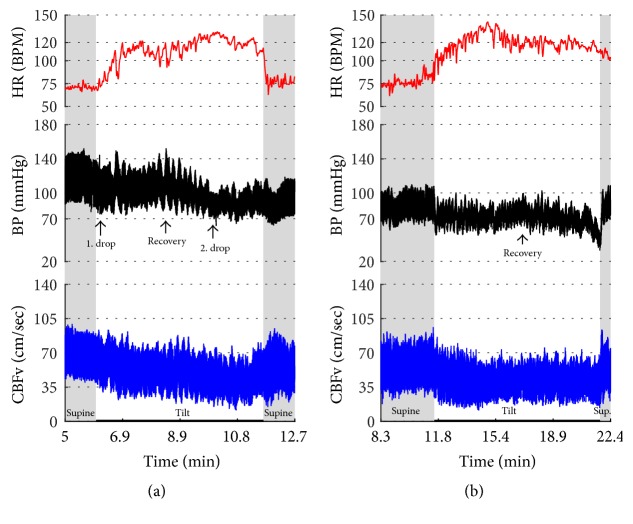
Orthostatic hypotension with reduced orthostatic CBFv. (a) shows the drop of BP (1. drop) at the beginning of the tilt with a recovery (recovery) followed by a further decline in BP at the second half of the tilt (2. drop). The HR increment was preserved during the tilt. CBFv was progressively declining during the tilt. Patient was very dizzy and anxious and she requested to terminate the tilt at the 6th minute. (b) shows data from the same person a year later. The second tilt test was remarkably similar to the first one showing orthostatic hypotension with similar recovery and final decline of the blood pressure. CBFv was reduced throughout the tilt. Data from 26-year-old woman.

**Figure 7 fig7:**
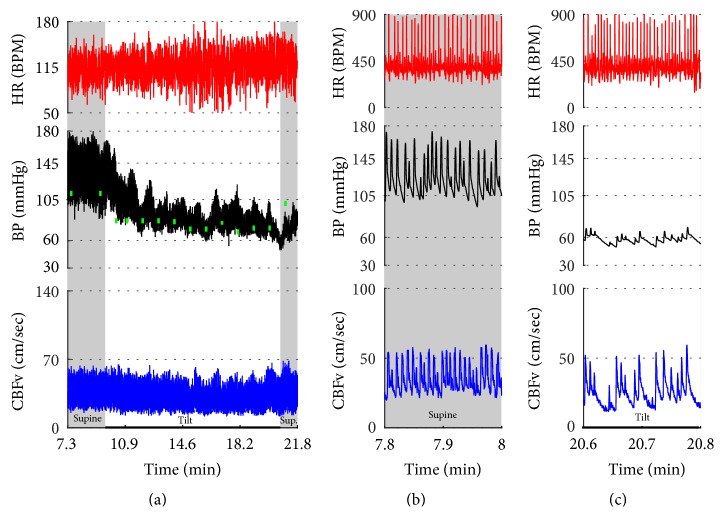
This example shows combination of (1) primary cerebral autoregulatory failure (pCAF); (2) severe orthostatic hypotension with reduced orthostatic CBFv; and (3) atrial fibrillation. Supine BP is elevated while supine CBFv is reduced. The pattern of elevated BP and reduced CBFv during supine position is due to abnormal cerebral vasoconstriction consistent with pCAF. There was severe OH with a progressive decrease in BP during the tilt. Diastolic CBFv was reduced during the tilt but less than systolic CBFv that can be seen in mild cerebral vasodilatation that compensates for reduced orthostatic BP. HR responses to the tilt were absent. Note random, noise-like pattern of HR due atrial fibrillation. The HR fluctuated wildly (50–180 BPM) and not all electrical systoles were transmitted in the mechanical systoles (c) resulting in marked variations of BP and CBFv. Supine hypertension and orthostatic hypotension are a marker of severe autonomic adrenergic failure. Green boxes represent mean blood pressure obtained from the arm. Data from 69-year-old man with multiple system atrophy.

**Figure 8 fig8:**
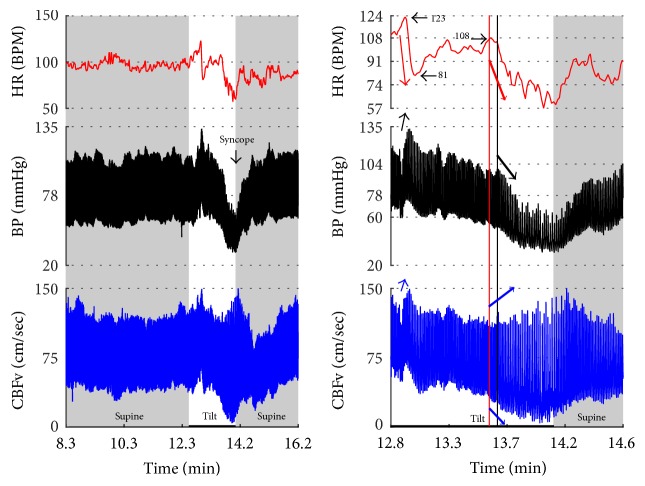
Mixed syncope. In this subject, initial slowing of HR from 123 to 81 BPM (thin red arrow) is not cardioinhibitory response but reflects a functioning baroreflex since it is associated with elevation of BP (thin black arrow) and CBFv (thin blue arrow). The onset of the cardioinhibitory reaction is marked by the vertical red line with HR 108 BPM (thick red arrow) and coincides with a BP drop (thick black arrow) and patient became quickly unconscious. The pulsatility index increased during syncope (systolic CBFv increased and diastolic CFBv decreased) which is consistent with cerebral vasodilatation. The vasodilatation started early (thick blue arrows), and the changes were discernible in the doppler audio signal before noticeable changes in heart rate or BP. The cardiac slowing followed with delayed BP decrease being characteristic of cardioinhibitory syncope. Patient lost consciousness when the systolic BP declined below 60 mmHg, as expected. Data from 20 y/o woman.

**Figure 9 fig9:**
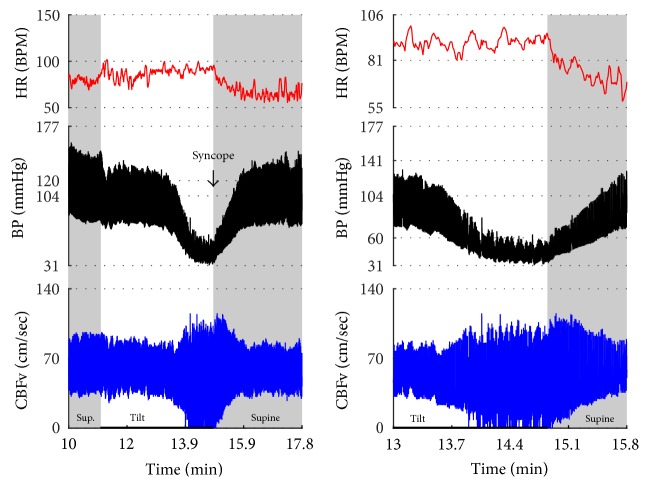
Vasodepressor syncope. There is a progressive drop in BP without bradycardia till syncope occurred. HR started to slow down only after a subject was tilted back to supine position. The syncope is associated with a characteristic cerebral vasodilatation pattern. Data from 27-year-old woman.

**Figure 10 fig10:**
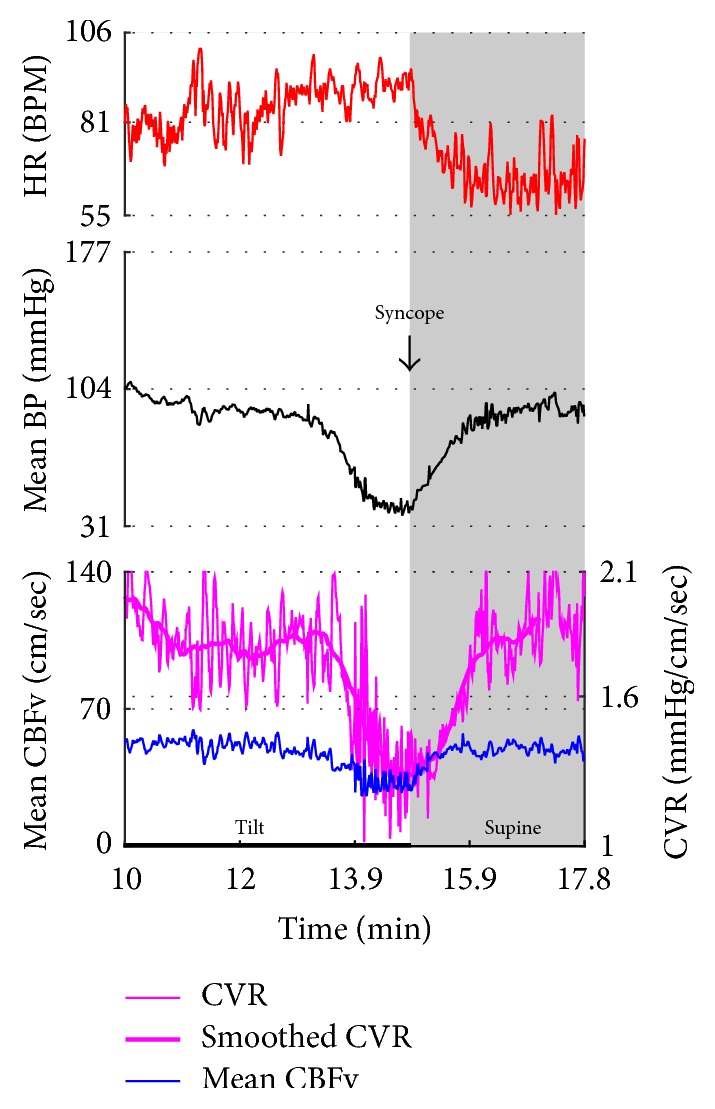
Cerebral vasodilation during syncope. The cerebral vascular resistance is reduced during syncope which is consistent with cerebral vasodilation. The same subject as in Figure 9.

**Figure 11 fig11:**
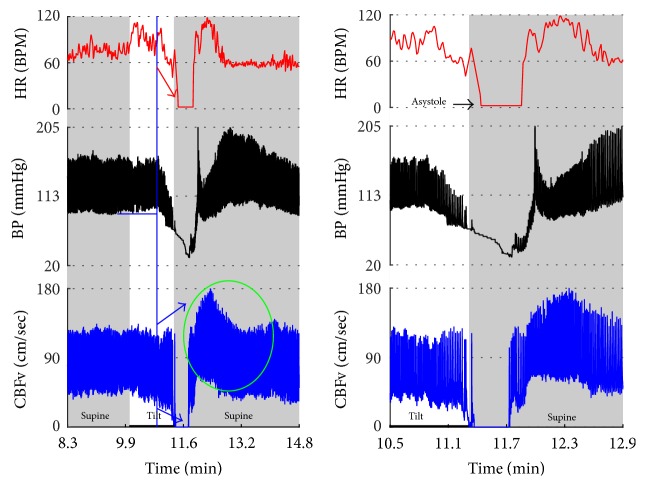
Cardioinhibitory syncope. In this subject, slowing of HR, BP drop, and cerebral vasodilation began almost simultaneously (marked by blue line) and progressed rapidly into asystole of 27 seconds. Note the vasodilatory pattern in CBFv early in the evolution of syncope (blue arrows) followed by reactive hyperemia (green circle) with elevated systolic and diastolic CBFv. Data from 39-year-old man.

OH can be progressive (Figures 1, 4, and 7), transient (Figure 5), associated with stable orthostatic CBFv, for example, compensated (Figures 4 and 5), or reduced orthostatic CBFv, for example, uncompensated (Figures 6 and 7). Mean blood pressure was reduced in both OH groups while the CBFV tilt drop score was abnormal in the OH-uncompensated group during the tilt (Table 2).

**Figure 12 fig12:**
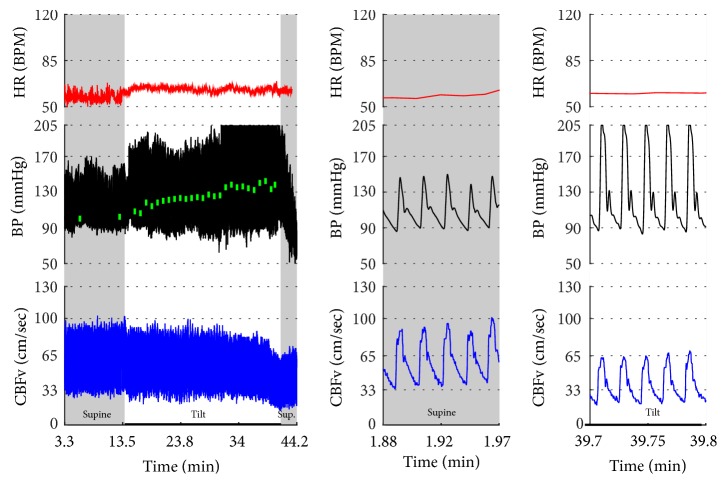
Orthostatic cerebral hypoperfusion syndrome (OCHOs). The tilt provoked a progressive increase in BP that was seen in both finger (black trace) and arm (green boxes) BP. The finger systolic BP exceeded the upper range of our device, 205 mmHg. The CBFv was progressively declining during the tilt that was consistent with cerebral vasoconstriction. Patient was very dizzy and agitated during the tilt. Data from 66-year-old woman.

OCHOs (Figures 1, 12, and 20) showed primary reduction in orthostatic CBFv with normal orthostatic heart rate responses and without orthostatic hypotension.

**Figure 13 fig13:**
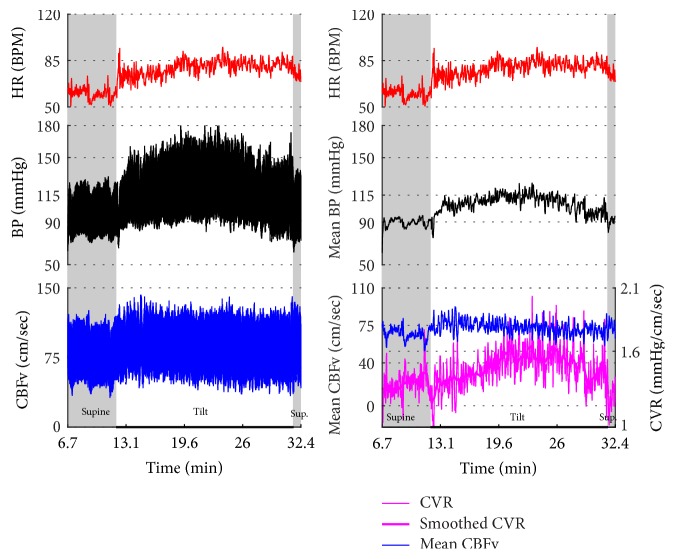
Orthostatic hypertension syndrome. BP was elevated during the tilt while CBFv was stable. HR responses were normal during the tilt. Note increased cerebral vascular resistance (CRV) that is consistent with cerebral vasoconstriction. Data from 39-year-old man.

**Figure 14 fig14:**
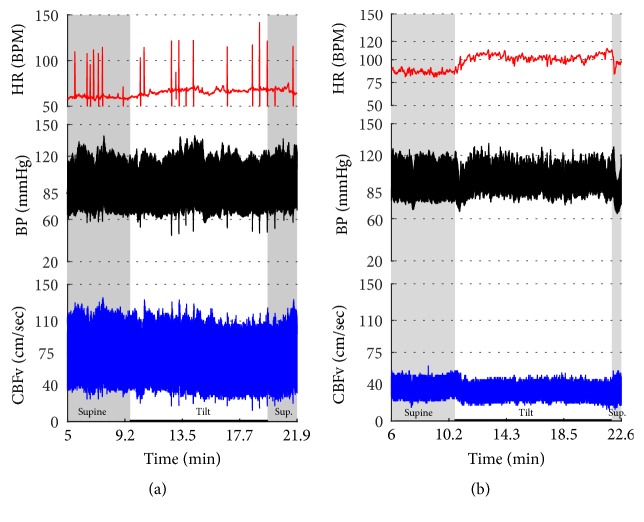
Primary cerebral autoregulatory failure (pCAF, (b)). HR and BP were normal at supine and during the tilt. CBFv was markedly reduced at the supine position and was further reduced during the tilt. Data from 47-year-old man. Patient was referred for evaluation of chronic fatigue, difficulites with attention, and chronic dizziness that was both supine and postural. For comparison, a healthy 80-year-old woman has normal CBFv (a).

**Figure 15 fig15:**
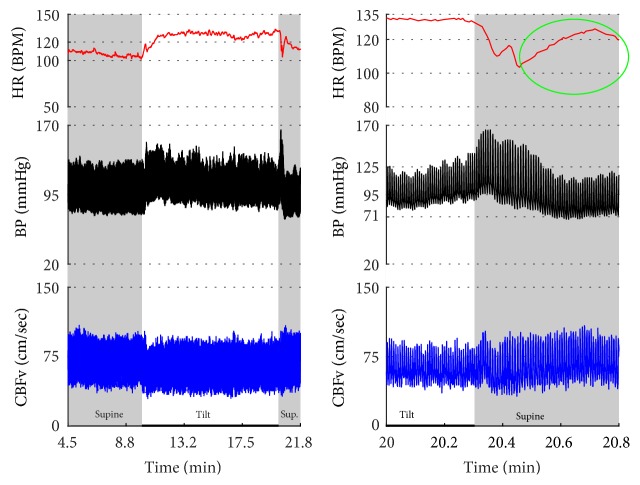
Inappropriate sinus tachycardia (IST). Note elevated resting heart rate (>100 BMP) which is further increased during the tilt. This response satisfies criteria for POTS (last baseline HR = 101.3, last HR during the tilt = 132.6, and increment > 30 BPM and BPM > 120) except that the continuous resting supine tachycardia is inconsistent with POTS. Second clue that this is not POTS is an episode of HR increment exceeding 120 BPM (green oval) at supine position after completing the tilt. Mean supine HR = 106.6 ± 2.8, range 97.1–114.3 BPM, mean orthostatic HR 128 ± 2.4, and range 121.8–134.0 BPM.

**Figure 16 fig16:**
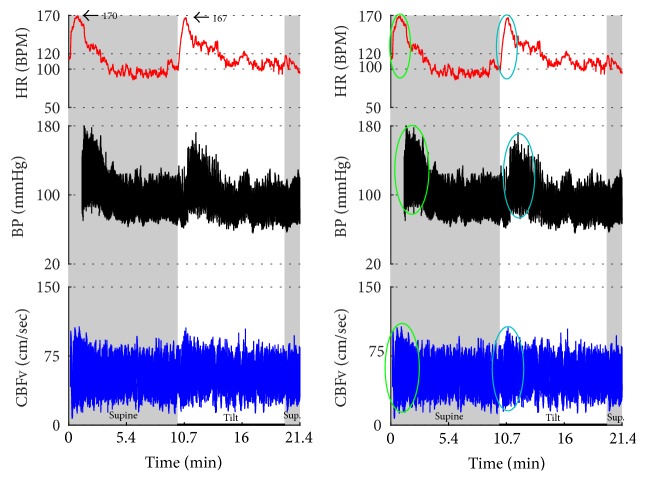
Paroxysmal sinus tachycardia (PST) due to anxiety. The patient, a 34 y/o woman, was referred for evaluation of postural tachycardia syndrome (POTS). During montage in supine position before placing the blood pressure sensor (i.e., why the initial portion of recording is missing), she became very anxious after she was informed that she will be tilted in several minutes. The anxiety was associated with transient tachycardia 170 BPM, elevated BP, and CBFv (green ovals). Similar pattern (transient tachycardia, elevated BP, and CBFv) was observed at the onset of the tilt (blue oval). The supine tachycardia of similar character to that of the tilt confirms that this is not POTS but IST. Furthermore, the tachycardia is usually sustained and/or it is progressively increased during the tilt in POTS. CBFv is usually unchanged or decreased in POTS during the tilt.

**Figure 17 fig17:**
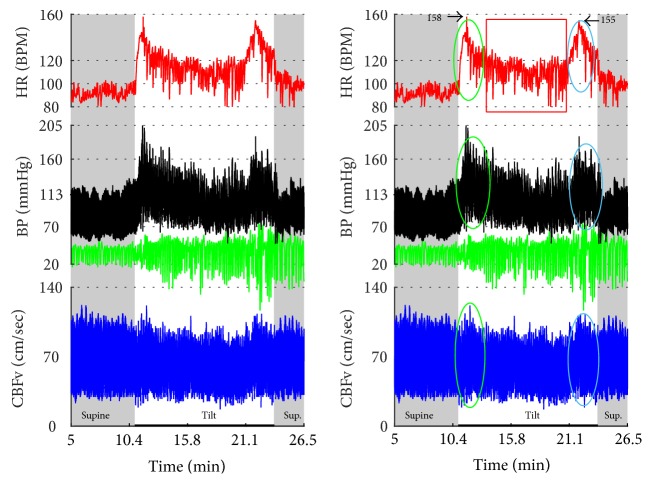
Postural tachycardia syndrome (POTS) or paroxysmal sinus tachycardia (PST) due to the anxiety reaction? The supine variables were normal. The tilt test induced initial tachycardia associated with elevation of BP and CBFv (green ovals). This reaction is due to anxiety since BP and CBFv are elevated. In POTS, CBFv is usually unchanged or reduced. Similar reaction occurred at the end of the tilt (blue ovals). Note that patient was hyperventilating during the tilt (green tracing). The tilt test was done without any medication. Trial of beta blockers failed to improve orthostatic intolerance. Final diagnosis was PST. Mean supine HR was 91.5 ± 4.9 BPM; mean orthostatic HR during more steady HR (demarcated by a red box) was 109.3 ± 8.5 BPM. The mean HR during the whole tilting was 118.5 ± 13.8 BPM. Data from 25-year-old woman.

In OHTN (Figure 13) orthostatic blood pressure was elevated while orthostatic CBFv was stable.

Characteristic features of tachycardia syndromes (POTS, IST, and PST) are shown in Figures 2 [Fig fig3] [Fig fig4] [Fig fig5] [Fig fig6] [Fig fig7] [Fig fig8] [Fig fig9] [Fig fig10] [Fig fig11] [Fig fig12] [Fig fig13] [Fig fig14], 15 [Fig fig16] [Fig fig17]–18, and 23 and Table 2. Both POTS and PST patients were younger than subjects with the normal tilt test and had elevated heart rate during the tilt. IST subjects were younger than subjects with the normal tilt response and had elevated heart rate in supine position and during the tilt.

**Figure 19 fig19:**
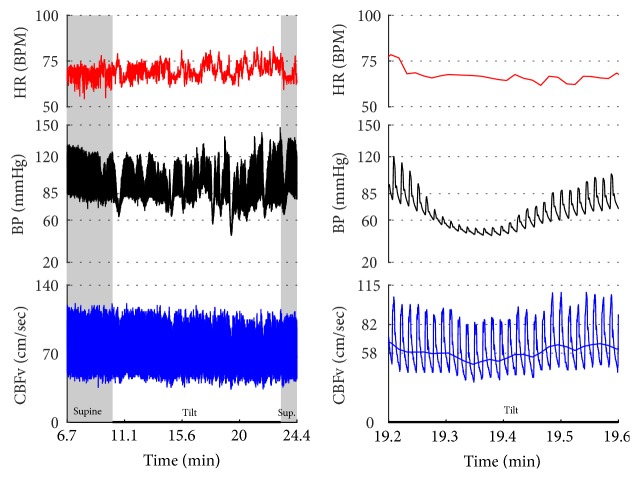
Vasomotor oscillations. Note marked fluctuations of BP occurring approximately every minute. CBFv also fluctuates in synchrony with BP. Patient was mildly anxious during the tilt test but she was not dizzy. Data from 47-year-old woman.

**Figure 20 fig20:**
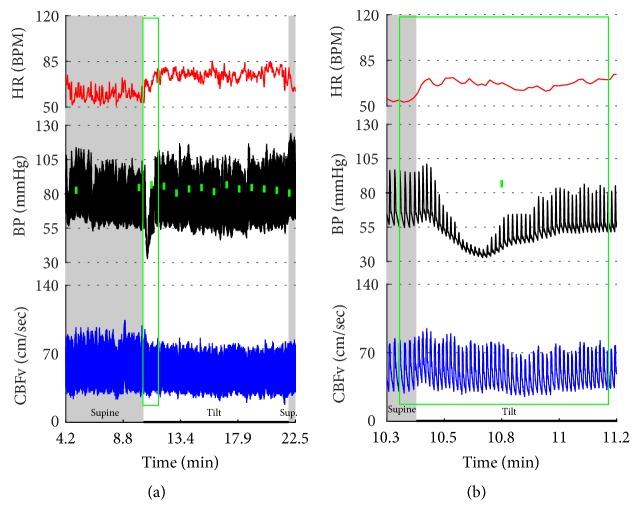
The transient drop in BP at the beginning of the tilt (designated by a green box) is artificial. The oscillometric device showed much higher BP (the small filled green box in (b)). Another clue that this is a technical artifact is the fact that there is no corresponding HR reaction and also CBFv is unchanged. The tilt test was normal. Data from 38-year-old woman.

**Figure 21:**
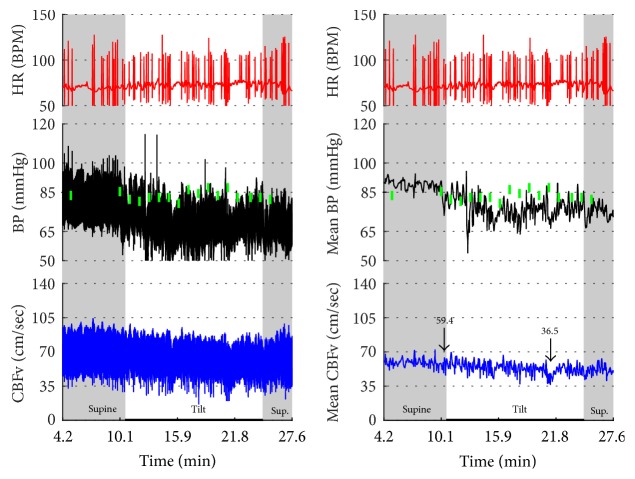
This example shows arteficial drift in the plethysmographic finger BP that may be misinterpreted as orthostatic hypotension. The spurious decline in BP continued in spite of multiple repositionings of a sensor. Note that oscillometric BP from the upper arm (green boxes) shows absent orthostatic hypotension. There are a number of extrasystoles in the HR. BP was interpreted as normal. CBFv responses to the tilt were abnormal as there was a drop in CBFv from the supine 59.4 cm/sec to 36.5 cm/sec at the end of the tilt. Patient was dizzy during the tilt. The final diagnosis was orthostatic cerebral hypoperfusion syndrome. Data from 43-year-old man.

Vasomotor oscillations (Figure 19), spurious fluctuations of blood pressure (Figure 20), and sustained drift in blood pressure (Figure 21) obtained from finger plethysmographic device can be recognized by simultaneous recording of the arm blood pressure and CBFv.

**Figure 22:**
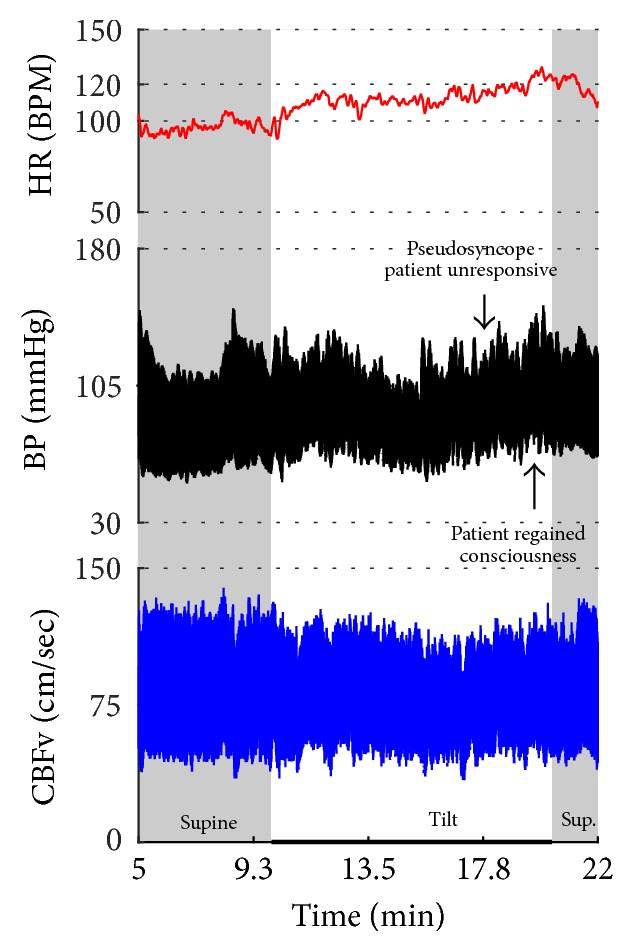
Psychogenic unresponsiveness. A patient became progressively obtunded and confused, experienced eyelid flutter, became aphasic, and finally did lose the consciousness during the tilt. The patient responded to reassurance and she regained consciousness while in the upright position. All monitoring variables were normal during the spell; patient became slightly tachycardiac after the spell. Data from 37-year-old woman.

**Figure 23:**
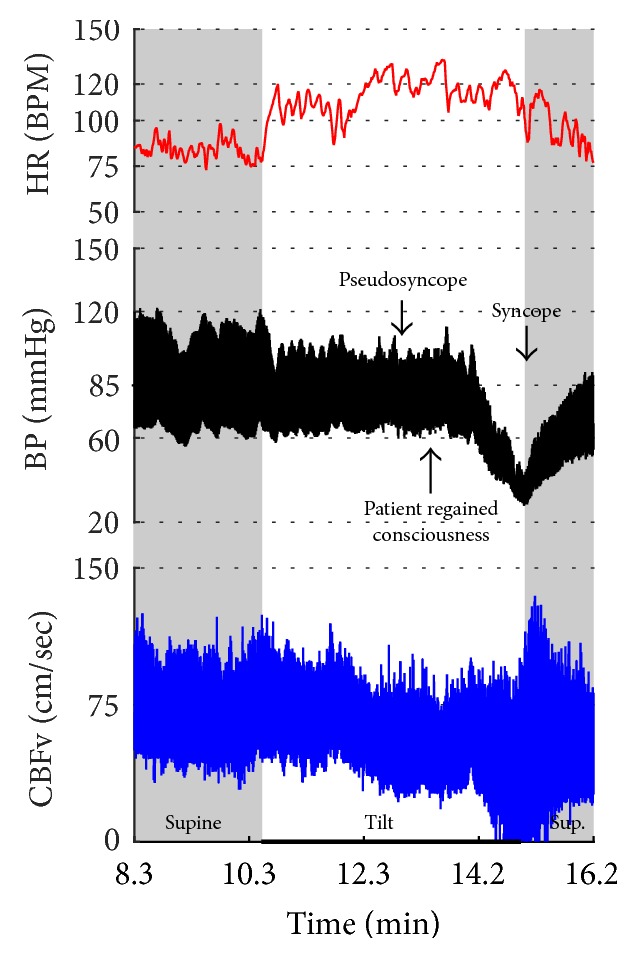
POTS + psychogenic unresponsiveness + syncope. The tilt provoked symptomatic excessive heart rate increment associated with a drop in CBFv without orthostatic hypotension which satisfies criteria for POTS. Then the patient became progressively less responsive which was consistent with pseudosyncope (psychogenic unresponsiveness) since CBFv was stable. The patient responded to reassurance and she regained consciousness later during the tilting. However, subsequently the patient experienced true mixed syncope at the 5th minute of the tilt (the tilt starts at minute 10.6 of the recording) and the tilt was terminated. Final diagnoses: (1) POTS; (2) psychogenic unresponsiveness; and (3) mixed syncope. Data from 21-year-old woman.

Eight patients had pseudosyncope (Figures 22-23) with stable orthostatic CBFv. From these patients, tilt test showed normal responses in 4 subjects, POTS in 2 subjects, and OH-compensated and mixed syncope in 1 subject. All these subjects regained consciousness during the tilt test by reassurance.

Patients with pCAF had reduced CBFv in the supine position, had elevated cerebral vascular resistance in supine position and during the tilt, and had abnormal drop of CBFv score during the tilt (Table 2).

Table 3 summarizes the main diagnostic features of common patterns encountered during the tilt test.

## 4. Discussion 

This study showed head-to-head comparisons of common tilt test patterns. Characteristic pattern associated with a particular orthostatic syndrome can be grouped into abnormalities predominantly affecting heart rate (PST, IST, and POTS), blood pressure (syncope, OH, and OHTN), and cerebral blood flow (OCHOs, pCAF). Psychogenic pseudosyncope is associated with stable CBFv without any particular heart rate or blood pressure pattern. This study also showed that criteria for several orthostatic syndromes had to be modified to allow unambiguous pattern classifications.

### 4.1. Symptoms versus Tilt Diagnosis

Orthostatic symptoms, except of syncope, are nonspecific and in general poorly correlating with orthostatic blood pressure or heart rate. This study showed that orthostatic drop in CBFv is more sensitive and specific marker of prediction of orthostatic symptoms than orthostatic blood pressure changes. Therefore CBFv is better proxy for cerebral hypoperfusion than orthostatic hypotension.

Criteria for orthostatic syndromes are heterogeneous; some criteria are exclusively physiological (e.g., OH which can be symptomatic or asymptomatic) while the others require also the presence of symptoms, for example, POTS. Nevertheless, the information obtained from the tilt test should not be used in isolation but always on clinical ground.

### 4.2. Tachycardia Syndromes (PST, IST, and POTS)

These syndromes share excessive heart rate increment. Primarily, it is the timing of tachycardia that distinguishes each syndrome. Using the nonmodified criteria, majority of patients with IST and PST satisfy also the POTS criteria. The ambiguities in determination of tachycardia syndromes were solved by modifying the diagnostic criteria as mentioned in the Section 2. CBFv is also helpful in classification. CBFv is characteristically reduced in POTS, normal in IST, and normal or transiently elevated in PST.

### 4.3. Neurally Mediated Syncope

Neurally mediated syncope has a characteristic pattern. Loss of consciousness usually occurs if systolic blood pressure declines to 60 mmHg [21] that is accompanied by cerebral vasodilation [15]. In this study, the CBFv pattern was similar in all types of syncope. The diastolic CBFv was close or equal to zero, the mean CBFv was less than 30 cm/sec, and pulsatility index increased which is consistent with cerebral vasodilation [15]. It is advantageous to use CBFv in differentiation of pseudosyncope or spurious decline in finger blood pressure; in both conditions CBFv remains stable.

### 4.4. Primary Cerebral Autoregulatory Failure (pCAF)

PCAF is defined as low supine CBFv without supine hypotension or other hemodynamic abnormalities that can explain low CBFv. Patients with pCAF have increased cerebral vascular resistance that may be due to cerebral autoregulatory failure [19]. CBFv is also reduced during the tilt in spite of stable orthostatic blood pressure, further pointing to altered cerebral autoregulation. It can be hypothesized that pCAF is associated with small vessel disease. This hypothesis is supported by a previous study that showed significant negative correlation between CBFv and severity of white matter abnormalities in the MRI which is a marker of small vessel disease [22]. Clinical significance of pCAF is unclear but pCAF may be a cause of cerebral dysfunction due to chronic cerebral hypoperfusion.

### 4.5. OHTN

Original definition of OHTN included only an increase of blood pressure during the tilt while heart rate and CBFv responses to tilting were not considered. These limitations can cause diagnostic overlap with POTS since occasional rise in blood pressure during tilting can be observed in the hyperadrenergic form of POTS [23]. Furthermore, the rise in blood pressure can be seen also in OCHOs. Consider an example in Figure 12, where the excessive rise in blood pressure is associated with drop in CBFv during the tilt test. This patient has diagnosis of OCHOs, but using the original OHTN criteria he also qualifies for diagnosis of OHTN. The above mentioned shortcomings were overcome by including the heart rate and CBFv responses in the OHTN definition (Table 1).

### 4.6. The Role of CBFv Monitoring during the Tilt Test

Typically, the tilt test uses heart rate and blood pressure responses in classification of results [24]. The usefulness of an additional CBFv monitoring during the tilt test can be demonstrated by comparing the yield of the tilt test with and without CBFv monitoring. Without CBFv and when using heart rate and blood pressure monitoring only, then all OCHOs subjects (13% or 97 subjects) will be labeled as having normal tilt test. Since they have orthostatic symptoms (100% of OCHOs patients were symptomatic in this study) they may be misdiagnosed as having psychogenic, vestibular, or an unclassified disorder. Furthermore 67 (9%) patients with diagnosis of pCAF would also be missed without using CBFv. CBFv also helps to differentiate PST, IST, and POTS and spurious blood pressure oscillations.

### 4.7. Pseudosyncope: Psychogenic Syncope

No specific changes in hemodynamic parameters except of stable CBFv have been found in this study. This is in contrast with a recent study [25] which observed elevated heart rate and blood pressure before and during the loss of consciousness. In this study no particular heart rate and blood pressure pattern could be detected. The difference in results may reflect different patient population evaluated in both studies. This study also found that pseudosyncope can be combined with other syndromes including syncope. CBFv monitoring appears to be invaluable in differentiation between pseudosyncope and syncope. The characteristic drop in CBFv associated with a vasodilatation pattern seen in syncope is missing in pseudosyncope.

### 4.8. Limitations of the Current Definitions of Orthostatic Syndromes

Ambiguities in definition of orthostatic syndromes were removed by adjusting the diagnostic criteria. Nevertheless, the clinical significance of adjusted criteria needs to be validated in future studies.

Table 3 summarizes common patterns associated with the tilt test and emphasizes the main features that enable differentiating each pattern.

## 5. Conclusion

Tilt test can be used as an add-on in diagnosis of orthostatic syndromes. However diagnostic criteria for several syndromes had to be made more explicit to allow unambiguous pattern classification. To be able to classify all patterns, it is essential to monitor CBFv in addition to blood pressure and heart rate.

## Figures and Tables

**Figure 2 fig2:**
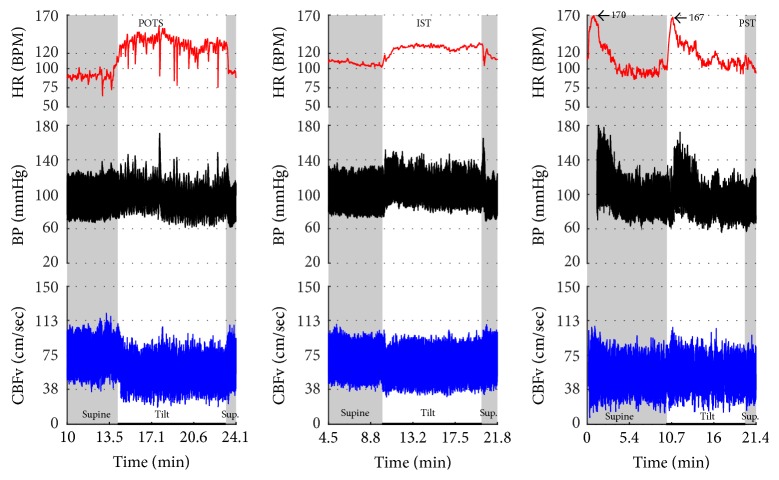
Comparisons of tachycardia syndromes including postural tachycardia syndrome (POTS), inappropriate sinus tachycardia (IST), and paroxysmal sinus tachycardia (PST). During supine position, the heart rate is normal (<100) in POTS, consistently elevated in IST, and transiently elevated in PST. The excessive tachycardia during the tilt is seen in all shown syndromes, being continuous in POTS and IST, and intermittent in PST. Blood pressure is stable in all examples. CBFv is reduced in POTS, normal in IST, and intermittently elevated in PST.

**Figure 18 fig18:**
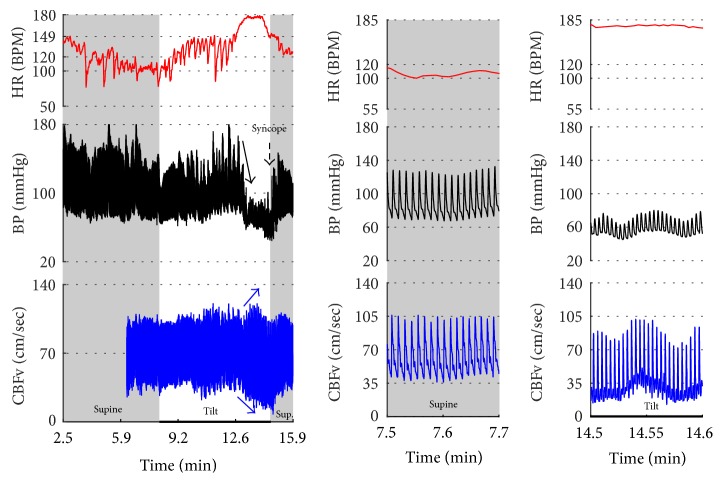
Inappropriate sinus tachycardia complicated by vasodepressor syncope. The average HR was above 100 BPM with the maximal heart rate 149 BPM during the supine position. The tilt provoked HR increase and marked oscillations in all signals including HR, BP, and CBFv. At the 5th minute of the tilt, BP suddenly declined (black arrow) that was accompanied by tachycardia 179 BPM culminated in a syncope. The decline in BP without bradycardia is characteristic of vasodepressor syncope due to reduced peripheral resistance. CBFv declined and the pulsatility index (systolic CBFv – diastolic CBFv) increased (blue arrows) that indicates cerebral vasodilation. Data from 26-year-old woman.

**Table 1:** Criteria for the orthostatic syndromes.

Disorder/syndrome	Criteria	Comments
Normal response to tilt	QASAT [1]—bradycardia supine = 0 AND QASAT [3]—increased heart rate response to tilt = 0 QASAT [7]—orthostatic hypotension during the tilt = 0 AND QASAT [10]—orthostatic hypertension during the tilt = 0 AND QASAT [15]—cerebral blood flow response to tilt = 0	

Orthostatic hypotension (OH), compensated, with stable orthostatic CBFv	QASAT [7]—orthostatic hypotension during the tilt > 0 AND QASAT [15]—cerebral blood flow response to tilt = 0	

OH, uncompensated, with reduced orthostatic CBFv	QASAT [7]—orthostatic hypotension during the tilt > 0 AND QASAT [15]—cerebral blood flow response to tilt > 0	

Orthostatic cerebral hypoperfusion syndrome (OCHOs)	QASAT [7]—orthostatic hypotension during the tilt = 0 AND QASAT [15]—cerebral blood flow response to tilt > 1 AND QASAT [3]—increased heart rate response to tilt = 0	

Orthostatic hypertension syndrome (OHTN)	QASAT [7]—orthostatic hypotension during the tilt = 0 AND QASAT [10]—orthostatic hypertension during the tilt = 1 AND QASAT [15]—cerebral blood flow response to tilt = 0 AND QASAT [3]—increased heart rate response to tilt = 0	

Postural tachycardia syndrome (POTS)	QASAT [7]—orthostatic hypotension during the tilt = 0 AND average supine heart rate before the tilt < 100 BMP AND maximal heart rate increment during the tilt ≥ 30 BMP AND the duration of the increment ≥ 3 minutes AND the maximal absolute heart rate during the increment ≥ 120 BPM	If the maximal heart rate does not cross 120 BMP, it is called mild orthostatic intolerance

Inappropriate sinus tachycardia (IST)	QASAT [7]—orthostatic hypotension during the tilt = 0 AND average supine heart rate before the tilt ≥ 100 BMP AND heart rate increment during the tilt ≥ 30 BMP	Grading is based on maximal heart rate

Paroxysmal sinus tachycardia (PST)	QASAT [7]—orthostatic hypotension during the tilt = 0 AND average supine heart rate before the tilt < 100 BMP AND maximal heart rate increment during the tilt ≥ 30 BMP AND the duration of the increment < 3 minutes AND the maximal absolute heart rate during the increment ≥ 120 BPM	PST usually affects the first 2 minutes of the tilt but not always. If, for example, the tachycardia with the 30 BPM increment occurs at minutes 9 and 10 of the tilt (the tachycardia duration = 2 minutes), then it is recommended to continue the tilt to clarify the diagnosis. If the tachycardia continues it is the POTS; if not it is PST

Syncope, cardiovagal	HR < 40 BPM AND systolic BP < 60 mmHg AND diastolic CBFv < 5 cm/sec	All variables are obtained during the syncope

Syncope, vasodepressor	HR ≥ before syncope AND systolic BP < 60 mmHg AND diastolic CBFv < 5 cm/sec	All variables are obtained during the syncope

Syncope, mixed	HR > 40 BPM AND HR < before syncope AND systolic BP < 60 mmHg AND diastolic CBFv < 5 cm/sec	All variables are obtained during the syncope

Primary cerebral autoregulatory failure	QASAT [13]—supine cerebral low flow = 1 AND QASAT [6]—supine hypotension = 0	

Psychogenic pseudosyncope	QASAT [15]—cerebral blood flow response to tilt = 0 AND physical examination indicative of apparent loss of consciousness	Typically patient is unresponsive, atonic, although bizarre posturing can be observed, without abnormal movement that can be seen in seizures

Comments: QASAT = Quantitative Scale for Grading of Cardiovascular Autonomic Reflex Tests and Small Fibers from Skin Biopsies, details of calculations can be found in Novak, 2015 [6]. HR = heart rate; BP = blood pressure; CBFv = cerebral blood flow velocity.

**Table 2:** Main clinical variables associated with orthostatic syndromes.

Diagnosis	New Dg	Age Gender, f/m BMI	OST	ROS	Baseline	Tilt	Comments
Normal *N* = 102	0	48.7 ± 16.7 64/38 26.9 ± 7.9	32	14	HR = 72.4 ± 14.3 BP = 92.2 ± 9.9 Q-LF = 0 ± 0 CVR = 1.5 ± 0.5	HR = 81.5 ± 13.9 BP = 92.3 ± 9.9 Q-TF = 0 ± 0 CVR = 1.6 ± 0.5	Orthostatic symptoms during the tilt test: dizziness or lightheadedness (2), palpitations (3), presyncope (patient believes is going to faint) 0, sense of weakness (3), shortness of breath (1), chest pain (0), excessive sweating (1), fatigue (4). 12 patients experienced dizziness associated with movement of the tilt table that resolved after the tilt table did reach the upright position.

OH-c *N* = 96	34	56.9 ± 17.5 59/37 27.7 ± 8.5	12	10	HR = 74.0 ± 7.2 BP = 95.5 ± 11.8 Q-LF = 0.2 ± 0.4	HR = 82.8 ± 19.8 BP = 85.6 ± 13.7^⋀^ Q-TF = 0.0 ± 0.0	Orthostatic symptoms during the tilt test: dizziness or lightheadedness (0), palpitations (0), presyncope (patient believes is going to faint) 0, sense of weakness (3), shortness of breath (3), chest pain (2), excessive sweating (1), fatigue (11).

OH-u *N* = 60	24	57.7 ± 17.6 37/23 26.8 ± 6.8	51	51	HR = 76.5 ± 5.4 BP = 97.9 ± 14.8^*∗*^ Q-LF = 0.5 ± 0.4	HR = 83.5 ± 19.8 BP = 73.8 ± 16.9^⋀^ Q-TF = 5.3 ± 2.6^*∗*^	Orthostatic symptoms during the tilt test: dizziness or lightheadedness (50), palpitations (33), presyncope (patient believes is going to faint) 37, sense of weakness (41), shortness of breath (14), chest pain (6), excessive sweating (33), fatigue (48).

OCHOs *N* = 97	97	48.1 ± 17.6 59/38 27.2 ± 5.9	97	95	HR = 74.8 ± 15.5 BP = 91.9 ± 12.2 Q-LF = 0.27 ± 0.46 CVR = 1.9 ± 0.9	HR = 91.3 ± 15.1 BP = 91.4 ± 11.7 Q-TF = 5.09 ± 2.3^*∗*^ CVR = 2.5 ± 1.0^*∗*^	Orthostatic symptoms during the tilt test: dizziness or lightheadedness (93), palpitations (12), presyncope (patient believes is going to faint) 44, sense of weakness (34), shortness of breath (6), chest pain (9), excessive sweating (22), fatigue (67).

OHTN *N* = 14	14	53.0 ± 16.1 3/11 28.1 ± 5.4	13	13	HR = 73.8 ± 11.2 BP = 89.4 ± 10.6 Q-LF = 0.16 ± 0.37 CVR = 1.4 ± 0.3	HR = 89.3 ± 20.7 BP = 95.7 ± 14.7^*∗*^ Q-TF = 1.4 ± 1.7 CVR = 1.7 ± 0.4	Orthostatic symptoms during the tilt test: dizziness or lightheadedness (4), palpitations (0), presyncope (patient believes is going to faint) 4, sense of weakness (10), shortness of breath (4), chest pain (3), excessive sweating (1), fatigue (12).

POTS *N* = 101	67	31.1 ± 9.7^⋀^ 86/15 23.9 ± 4.7	100	100	HR = 87.1 ± 11.2 BP = 92.8 ± 11.7 Q-LF = 0.06 ± 0.24	HR = 143.0 ± 14.5^*∗*^ BP = 84.9 ± 18.8 Q-TF = 3.6 ± 3.7^*∗*^	Orthostatic symptoms during the tilt test: dizziness or lightheadedness (82), palpitations (80), presyncope (patient believes is going to faint) 62, sense of weakness (72), shortness of breath (30), chest pain (31), excessive sweating (18), fatigue (34). HR during the tilt is the average of maximal values of each subject.

IST *N* = 28	24	33.5 ± 7.5^⋀^ 22/6 26.6 ± 6.7	24	24	HR = 110.2 ± 7.4^*∗*^ BP = 97.7 ± 9.8 Q-LF = 0 ± 0	HR = 133.8 ± 18.3^*∗*^ BP = 94.5 ± 13.5 Q-TF = 0.0 ± 0.0	Orthostatic symptoms during the tilt test: dizziness or lightheadedness (21), palpitations (24), presyncope (patient believes is going to faint) 12, sense of weakness (15), shortness of breath (18), chest pain (9), excessive sweating (8), fatigue (18).

PST *N* = 12	12	37 ± 17.8^⋀^ 10/2 25.1 ± 4.8	12	12	HR = 82.0 ± 10.1 BP = 97.6 ± 12.9 Q-LF = 0 ± 0	HR = 134.5 ± 16.7^*∗*^ BP = 99.8 ± 11.6 Q-TF = 0.0 ± 0.0	Orthostatic symptoms during the tilt test: dizziness or lightheadedness (3), palpitations (12), presyncope (patient believes is going to faint) 2, sense of weakness (5), shortness of breath (7), chest pain (1), excessive sweating (2), fatigue (10). HR during the tilt is the average of maximal values of each subject.

Syncope-cardioinhibitory *N* = 4	3	39.0 ± 14.5 3/1 26.3 ± 6.2	4	3	HR = 70.0 ± 14.4 BP = 86.1 ± 11.5 Q-LF = 0 ± 0	HR = 12.1 ± 12.5^⋀^ BP = 40.3 ± 8.7^⋀^ CBFv = 22.3 ± 11.5^⋀^	Loss of consciousness (4), additional orthostatic symptoms during the tilt test: dizziness or lightheadedness (3), palpitations (0), presyncope (patient believes is going to faint) 4, sense of weakness (3), shortness of breath (4), chest pain (0), excessive sweating (2), fatigue (1). Tilt vitals obtained during syncope.

Syncope-vasodepressor *N* = 12	11	48.3 ± 22.5 7/5 26.4 ± 6.2	12	2	HR = 80.1 ± 16.7 BP = 90.7 ± 19.1 Q-LF = 0 ± 0	HR = 97.1 ± 21.2 BP = 36.7 ± 5.6^⋀^ CBFv = 26.6 ± 5.8^⋀^	Loss of consciousness (12); additional orthostatic symptoms during the tilt test: dizziness or lightheadedness (11), palpitations (7), presyncope (patient believes is going to faint) 8, sense of weakness (8), shortness of breath (9), chest pain (1), excessive sweating (7), fatigue (4). Tilt vitals obtained during syncope.

Syncope-mixed *N* = 26	10	35.2 ± 17.1^⋀^ 14/12 26.3 ± 10.9	26	12	HR = 77.8 ± 10.6 BP = 89.9 ± 12.1 Q-LF = 0 ± 0	HR = 66.5 ± 4.9^⋀^ BP = 41.8 ± 5.6^⋀^ CBFv = 28.1 ± 10.3^⋀^	Loss of consciousness (26); additional orthostatic symptoms during the tilt test: loss of consciousness (22), dizziness or lightheadedness (21), palpitations (14), presyncope (patient believes is going to faint) 22, sense of weakness (25), shortness of breath (17), chest pain (2), excessive sweating (21), fatigue (19). Tilt vitals obtained during syncope.

pCAF *N* = 67	67	57.9 ± 17.9 31/36 28.4 ± 6.7^*∗*^	22	22	HR = 72.2 ± 13.1 BP = 96.7 ± 14.8 Q-LF = 1 ± 0^*∗*^ CVR = 2.9 ± 0.8^*∗*^	HR = 82.2 ± 15.7 BP = 87.8 ± 16.7 Q-TF = 5.4 ± 2.7^⋀^ CVR = 4.1 ± 1.1^*∗*^	Orthostatic symptoms during the tilt test: dizziness or lightheadedness (61), palpitations (10), presyncope (patient believes is going to faint) 33, sense of weakness (45), shortness of breath (17), chest pain (12), excessive sweating (21), fatigue (50). All patients had also prominent nonpositional symptoms including chronic fatigue (33), weakness (32), forgetfulness (45), and attention deficit (54).

Psychogenic pseudosyncope *N* = 8	3	53.6 ± 19.9 7/1 28.8 ± 6.4	8	8	HR = 77.3 ± 15.1 BP = 95.3 ± 8.6 Q-LF = 0 ± 0	HR = 88.6 ± 17.8 BP = 93.7 ± 27.5 Q-TF = 0 ± 0	Loss of consciousness (8); additional orthostatic symptoms during the tilt test: dizziness or lightheadedness (6), palpitations (4), presyncope (patient believes is going to faint) 2, sense of weakness (4), shortness of breath (5), chest pain (1), excessive sweating (2), fatigue (5), eye flutter (4), moaning (4), bizarre posturing (4). Tilt vitals obtained during pseudosyncope.

Comments: diagnosis = diagnosis obtained during the tilt test; new Dg = diagnosis obtained from the tilt test that is different from the referral diagnosis or it is not documented in the chart (e.g., if the patient was referred for evaluation of orthostatic hypotension and the tilt test showed POTS); *N* = number of subjects; OST = orthostatic symptoms during the tilt, the number of subjects with at least one orthostatic symptom; ROS = reproduction of previous orthostatic symptoms (e.g., the symptoms that prompted the autonomic testing) during the tilt; OH = orthostatic hypotension; OH-compensated = OH with normal orthostatic CBFv; OH-uncompensated = OH with reduced orthostatic CBFv; OCHOs = orthostatic cerebral hypoperfusion syndrome; OHTN = orthostatic hypertension syndrome; POTS = postural tachycardia syndrome; IST = inappropriate sinus tachycardia; PST = paroxysmal sinus tachycardia; pCAF = primary cerebral autoregulatory failure; BMI = body mass index (kg/m^2^); CBFv = cerebral blood flow velocity (cm/s); HR = heart rate (beats per minute), BP = blood pressure (mmHg); Q-LF = QASAT supine low CBFv score, Q-TF = QASAT CBFv during the tilt score. All variables expressed as mean ± sd if not described otherwise; ^*∗*^elevated compared to normals; ^⋀^reduced compared to normal response to the tilt.

**Table 3:** The characteristics of the patterns associated with the tilt test.

Disorder/syndrome	HR		BP		CBFv		ET_CO_2__		Comments
	Supine	Tilt	Supine	Tilt	Supine	Tilt	Supine	Tilt	
Orthostatic hypotension (OH), compensated, with stable orthostatic CBFv	*↔*	↕	↕	↓^*∗*^	*↔*	*↔*	*↔*	*↔*	OH with stable orthostatic CBFv indicating preserved cerebral autoregulation. OH can have any pattern: early, late, sustained, intermittent, progressive, stable. Patients are typically not dizzy during the tilting

OH, uncompensated, with reduced orthostatic CBFv	*↔*	↕	↕	↓^*∗*^	*↔*	↓	*↔*	*↔*	OH with reduced orthostatic CBFv indicating either autoregulatory failure or BP below the autoregulatory range. Patients are typically dizzy during the tilting

Orthostatic cerebral hypoperfusion syndrome (OCHOs)	*↔*	*↔*	↕	*↔*	*↔*	↓^*∗*^	*↔*	*↔*	Orthostatic CBFv is low without OH or arrhythmia. Patients are typically dizzy during the tilting

Orthostatic hypertension syndrome (OHTN)	*↔*	*↔*	↕	↑^*∗*^	*↔*	*↔*↑	*↔*	*↔*	If HR increases ≥30 BPM during the tilt then it is POTS. If HR increases <30 BPM and CBFv decreases during the tilt then it is OCHOs

Postural tachycardia syndrome (POTS)	*↔*	↑^*∗*^	*↔*↓	*↔*↑	*↔*	*↔*↓	*↔*	*↔* or HV	HR increase during the tilt is usually sustained. HR ≥ 120 BPM during the tilt is also required. If maximal HR < 120 BPM during the tilt then it is called “mild orthostatic intolerance”

Inappropriate sinus tachycardia (IST)	↑^*∗*^	↑^*∗*^	*↔*↑	*↔*↑	*↔*	*↔*	*↔*	*↔*	HR during the supine may fluctuate but mean HR > 100 BPM during supine. HR is increased during the tilt and the increase is usually fluctuating

Paroxysmal sinus tachycardia (PST)	*↔*↑	↑^*∗*^	↕	*↔*↑	*↔*	*↔*↑	*↔* or HV	*↔* or HV	HR increase which may happen during both supine and the tilt is usually intermittent and associated with anxiety and may respond to reassurance

Syncope, cardiovagal	*↔*	↓	*↔*	↓^*∗*^	*↔*	↓	*↔*	↕	HR < 40 BPM

Syncope, vasodepressor	*↔*	*↔*↑	*↔*	↓^*∗*^	*↔*	↓	*↔*	↕	Minimal HR slowing (<10%)

Syncope, mixed	*↔*	↓	*↔*	↓^*∗*^	*↔*	↓	*↔*	↕	Both HR and BP decrease

Primary cerebral autoregulatory failure (pCAF)	*↔*	*↔*	*↔*↑	*↔*	↓^*∗*^	*↔*↓	*↔*	*↔*	Hyperventilation should be ruled out

Psychogenic pseudosyncope	↕	↕	↕	↕	↔^*∗*^	↔^*∗*^	↕	↕	Pseudosyncope can occur in both supine and upright position

Comments: CBFv = cerebral blood flow velocity; ET_CO_2__ = end tidal CO_2_; HR = heart rate; BP = blood pressure; BPM = beats per minute; supine = absolute values of respective variables in supine position; tilt = absolute values of respective variables during the tilt (upright position); HV = hyperventilation defined as ET_CO_2__ < 35 mmHg in supine position and < 30 mmHg during the tilt; CAF = cerebral autoregulatory failure; ↑ = increased; ↓ = decreased; ↕ = any; *↔* normal; *↔*↑ = normal or increased; *↔*↓ = normal or decreased. *∗* indicates the primary abnormality.

## References

[1] Smith J. J., Porth C. M., Erickson M. (1994). Hemodynamic response to the upright posture. *Journal of Clinical Pharmacology*.

[2] Kenny R. A., Ingram A., Bayliss J., Sutton R. (1986). Head-up tilt: a useful test for investigating unexplained syncope. *The Lancet*.

[3] Moya A., Sutton R., Ammirati F. (2009). Guidelines for the diagnosis and management of syncope (version 2009). *European Heart Journal*.

[4] Novak V., Spies J. M., Novak P., McPhee B. R., Rummans T. A., Low P. A. (1998). Hypocapnia and cerebral hypoperfusion in orthostatic intolerance. *Stroke*.

[5] Novak P. (2011). Quantitative autonomic testing. *Journal of Visualized Experiments*.

[6] Novak P. (2015). Quantitative scale for grading of cardiovascular autonomic reflex tests and small fibers from skin biopsies (QASAT). *Journal of Neurological Disorders*.

[7] Sheldon R. S., Grubb B. P., Olshansky B. (2015). 2015 Heart rhythm society expert consensus statement on the diagnosis and treatment of postural tachycardia syndrome, inappropriate sinus tachycardia, and vasovagal syncope. *Heart Rhythm*.

[8] Low P. A., Schondorf R., Novak V., Sandroni P., Opfer-Gehrking T. L., Novak P., Low P. A. (1997). Postural tachycardia syndrome. *Clinical Autonomic Disorders*.

[9] Plash W. B., Diedrich A., Biaggioni I. (2013). Diagnosing postural tachycardia syndrome: comparison of tilt testing compared with standing haemodynamics. *Clinical Science*.

[10] Wu J.-S., Yang Y.-C., Lu F.-H., Wu C.-H., Chang C.-J. (2008). Population-based study on the prevalence and correlates of orthostatic hypotension/hypertension and orthostatic dizziness. *Hypertension Research*.

[11] Fan X.-H., Sun K., Zhou X.-L., Zhang H.-M., Wu H.-Y., Hui R.-T. (2011). Association of orthostatic hypertension and hypotension with target organ damage in middle and old-aged hypertensive patients. *Zhonghua Yi Xue Za Zhi*.

[12] Fessel J., Robertson D. (2006). Orthostatic hypertension: when pressor reflexes overcompensate. *Nature Clinical Practice Nephrology*.

[13] Wieling W., Jardine D. L., de Lange F. J. (2016). Cardiac output and vasodilation in the vasovagal response: an analysis of the classic papers. *Heart Rhythm*.

[14] Wieling W., Jardine D. L., De Lange F. J. (2016). Cardiac output and vasodilation in the vasovagal response: an analysis of the classic papers. *Heart Rhythm*.

[15] Ocon A. J., Messer Z., Medow M. S., Stewart J. M. (2011). Increased pulsatile cerebral blood flow, cerebral vasodilation, and postsyncopal headache in adolescents. *The Journal of Pediatrics*.

[16] Brignole M., Menozzi C., Del Rosso A. (2000). New classification of haemodynamics of vasovagal syncope: beyond the VASIS classification. Analysis of the pre-syncopal phase of the tilt test without and with nitroglycerin challenge. Vasovagal Syncope International Study. *Europace*.

[17] Yusuf S., Camm A. J. (2005). The sinus tachycardias. *Nature Clinical Practice Cardiovascular Medicine*.

[18] Olshansky B., Sullivan R. M. (2013). Inappropriate sinus tachycardia. *Journal of the American College of Cardiology*.

[19] Novak P. (2016). Orthostatic cerebral hypoperfusion syndrome. *Frontiers in Aging Neuroscience*.

[20] Novak V., Novak P., Schondorf R. (1994). Accuracy of beat-to-beat noninvasive measurement of finger arterial pressure using the Finapres: a spectral analysis approach. *Journal of Clinical Monitoring*.

[21] Sheldon R., Killam S. (1992). Methodology of isoproterenol-tilt table testing in patients with syncope. *Journal of the American College of Cardiology*.

[22] Novak V., Last D., Alsop D. C. (2006). Cerebral blood flow velocity and periventricular white matter hyperintensities in type 2 diabetes. *Diabetes Care*.

[23] Low P. A., Sandroni P., Joyner M., Shen W.-K. (2009). Postural tachycardia syndrome (POTS). *Journal of Cardiovascular Electrophysiology*.

[24] Saal D. P., Thijs R. D., Van Dijk J. G. (2016). Tilt table testing in neurology and clinical neurophysiology. *Clinical Neurophysiology*.

[25] Tannemaat M. R., Van Niekerk J., Reijntjes R. H., Thijs R. D., Sutton R., Van Dijk J. G. (2013). The semiology of tilt-induced psychogenic pseudosyncope. *Neurology*.

